# The first multicentre study on coronary anomalies in the Netherlands: MuSCAT

**DOI:** 10.1007/s12471-021-01556-9

**Published:** 2021-03-08

**Authors:** C. J. Koppel, B. W. Driesen, R. J. de Winter, A. E. van den Bosch, R. van Kimmenade, L. J. Wagenaar, J. W. Jukema, M. G. Hazekamp, F. van der Kley, M. R. M. Jongbloed, P. Kiès, A. D. Egorova, D. B. H. Verheijen, P. Damman, P. H. Schoof, J. Wilschut, M. Stoel, R. G. H. Speekenbrink, M. Voskuil, H. W. Vliegen

**Affiliations:** 1 Department of Cardiology, CAHAL, Centre for Congenital Heart Disease Amsterdam-Leiden, Leiden University Medical Centre, Leiden, The Netherlands; 2 Department of Cardiology, University Medical Centre Utrecht, Utrecht, The Netherlands; 3 Department of Cardiology, CAHAL, Centre for Congenital Heart Disease Amsterdam-Leiden, Amsterdam University Medical Centres, location AMC, Amsterdam Zuidoost, The Netherlands; 4 Department of Cardiology, Erasmus Medical Centre, Rotterdam, The Netherlands; 5 Department of Cardiology, Radboud University Medical Centre, Nijmegen, The Netherlands; 6 Thorax Centre Twente, Medisch Spectrum Twente, Enschede, The Netherlands; 7 Department of Cardiothoracic Surgery, Leiden University Medical Centre, Leiden, The Netherlands; 8 Department of Anatomy and Embryology, Leiden University Medical Centre, Leiden, The Netherlands; 9 Department of Cardiothoracic Surgery, Universitair Medisch Centrum Utrecht, Utrecht, The Netherlands

**Keywords:** Coronary anomalies, Anomalous coronary artery from the opposite sinus of Valsalva, Coronary arteriovenous fistula, Anomalous coronary artery from the pulmonary artery, Multicentre study

## Abstract

**Background:**

Current guidelines on coronary anomalies are primarily based on expert consensus and a limited number of trials. A gold standard for diagnosis and a consensus on the treatment strategy in this patient group are lacking, especially for patients with an anomalous origin of a coronary artery from the opposite sinus of Valsalva (ACAOS) with an interarterial course.

**Aim:**

To provide evidence-substantiated recommendations for diagnostic work-up, treatment and follow-up of patients with anomalous coronary arteries.

**Methods:**

A clinical care pathway for patients with ACAOS was established by six Dutch centres. Prospectively included patients undergo work-up according to protocol using computed tomography (CT) angiography, ischaemia detection, echocardiography and coronary angiography with intracoronary measurements to assess anatomical and physiological characteristics of the ACAOS. Surgical and functional follow-up results are evaluated by CT angiography, ischaemia detection and a quality-of-life questionnaire. Patient inclusion for the first multicentre study on coronary anomalies in the Netherlands started in 2020 and will continue for at least 3 years with a minimum of 2 years of follow-up. For patients with a right or left coronary artery originating from the pulmonary artery and coronary arteriovenous fistulas a registry is maintained.

**Results:**

Primary outcomes are: (cardiac) death, myocardial ischaemia attributable to the ACAOS, re-intervention after surgery and intervention after initially conservative treatment. The influence of work-up examinations on treatment choice is also evaluated.

**Conclusions:**

Structural evidence for the appropriate management of patients with coronary anomalies, especially (interarterial) ACAOS, is lacking. By means of a structured care pathway in a multicentre setting, we aim to provide an evidence-based strategy for the diagnostic evaluation and treatment of this patient group.

**Supplementary Information:**

The online version of this article (10.1007/s12471-021-01556-9) contains supplementary material, which is available to authorized users.

## Introduction

Coronary anomalies are rare and often an incidental finding in patients without concomitant congenital heart defects. Conflicting reports and lack of evidence-substantiated guidelines lead to challenging treatment decisions, especially for anomalous coronary arteries originating from the opposite sinus of Valsalva or from the opposite coronary artery (ACAOS). For an anomalous coronary artery from the pulmonary artery or coronary arteriovenous fistula (CAVF) there is a more established consensus on treatment, but results of and recommendations on long-term follow-up are scarce[1, 2]. American and European guidelines both state that there is limited evidence regarding treatment choices, and the majority of their treatment advice is based on level C evidence [1, 3]. This underlines the gap in our knowledge concerning these defects and the need for more evidence.

## Anomalous coronary artery from the opposite sinus

**Fig. 1:**
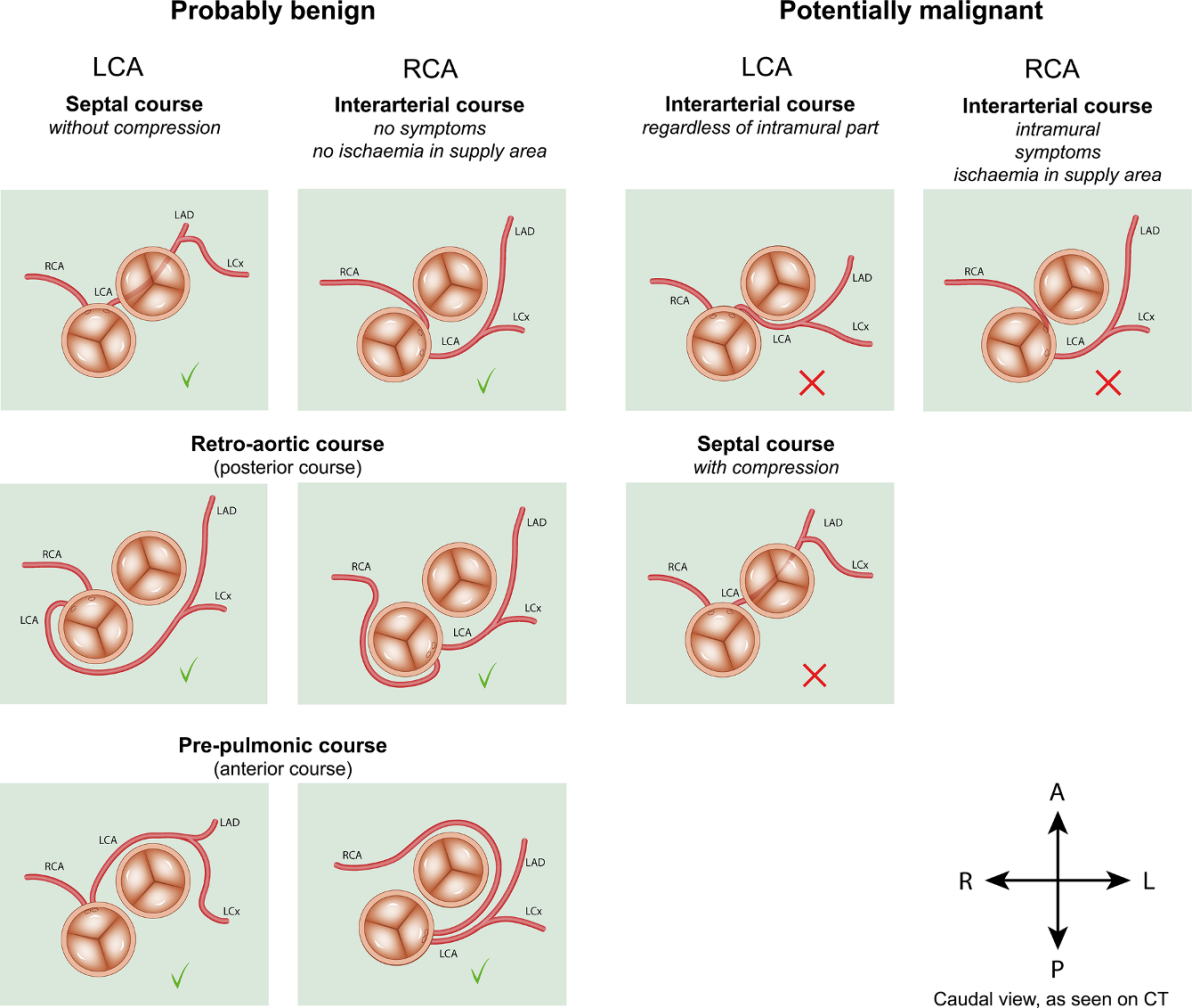
Variations of anomalous coronary arteries from the opposite sinus of Valsalva. *LCA* left coronary artery, *RCA* right coronary artery, *LAD* left anterior descending artery, *LCx* left circumflex artery, *R* right, *L* left, *A* anterior, *P* posterior, *CT* computed tomography

The prevalence of ACAOS is reported to be between 0.1 and 1% in the general population [4]. Multiple variations exist, which are divided into potentially benign and malignant variants (Fig. 1). Each variation demands a different approach in medical practice [4, 5]. The most important potentially malignant variation is an interarterial course, which can be a significant risk factor for sudden cardiac death (SCD) in physically active patients [6–8]. Several high-risk anatomical features potentially contribute to the risk of ischaemia and SCD: a (long) intramural course (where the coronary artery shares its tunica media with the aorta), a slit-like ostium, proximal narrowing, an elliptical vessel shape, acute angle take-off and dominance of the ACAOS [4, 5, 9, 10]. The precise mechanism causing ischaemia in interarterial ACAOS is not known. The reported risk of SCD is higher for an interarterial left coronary artery (LCA) than for the more prevalent interarterial right coronary artery (RCA) [4–6, 9, 10].

Evidence to guide treatment choices is currently scarce. Guidelines are primarily based on expert consensus and the findings of a limited number of studies [1, 3]. A class I indication for surgery exists only for ACAOS cases with an interarterial course and clear ischaemia-related complaints or proven ischaemia in the vessel’s supply area. However, age can be a complicating factor in this seemingly straightforward advice. In patients > 35 years, causes other than the ACAOS are seen to provoke complaints and ischaemia, such as obstructive or microvascular coronary artery disease or pulmonary disease, so for these patients treatment choices are not univocal [5, 11, 12]. Older patients also seem less prone to cardiac events caused by an interarterial ACAOS, for which reasons have not yet been elucidated [5]. For interarterial ACAOS without evidence of ischaemia, the American Heart Association (AHA)/American College of Cardiology (ACC) and the European Society of Cardiology (ESC) both provide a class IIa or lower level of evidence for surgical intervention. The AHA/ACC and ESC differ in their opinions on one point: the asymptomatic right interarterial ACAOS without myocardial ischaemia and without high-risk anatomy. The ESC specifically does not recommend (class III indication) surgery in these patients [3], but the AHA/ACC is not unequivocal with regard to surgical treatment versus continued observation (class IIb) [1]. Important when deciding on surgery are the expected benefits from surgery versus the peri-operative risks and risk of SCD of the uncorrected ACAOS. The methods most often applied for surgical correction are unroofing (preferred procedure in patients with a long intramural course), ostioplasty, and re-implantation of the coronary artery. Recent studies show that surgery is a safe option [4, 13, 14].

The beneficial effects of lifestyle restrictions and medical therapy (usually beta blockers) for a malignant ACAOS remain unclear and may contribute to anxiety regarding which level of exercise is considered safe [1, 4, 9]. For benign anatomical variants the consensus is that no interventions, exercise restrictions or medication are indicated [4].

In summary, the decision regarding how aggressive the approach to treat an ACAOS should be largely depends on the degree of malignant anatomical and physiological characteristics of the artery, i.e. the likelihood that the ACAOS causes ischaemia and/or a concomitant increased risk of SCD (Fig. 1). In clinical practice the criteria to decide whether an ACAOS is a benign or malignant variant are not uniform, and a ‘gold standard approach’ is lacking. Coronary computed tomography (CT) angiography is the best method to define the coronary anatomy and aids in the evaluation of anatomical high-risk features [1, 5], but physiological consequences cannot be adequately assessed with this technique. The most frequently used examinations to evaluate physiological aspects are: coronary angiography (CAG) with intracoronary measurements [15], stress echocardiography, exercise stress testing, perfusion magnetic resonance imaging with adenosine and nuclear perfusion imaging. These tests are endorsed by the 2018 AHA/ACC and 2020 ESC guidelines for the management of adults with congenital heart disease (class I indication) [1, 3], but it is unclear which examination(s) provide(s) the physician with the most useful information regarding the treatment decision. As a result, current clinical practice is very heterogeneous.

## Aberrant left or right coronary artery from pulmonary artery

It is recommended that an aberrant left coronary artery from the pulmonary artery (ALCAPA) and an aberrant right coronary artery from the pulmonary artery (ARCAPA) are corrected because of the risk of myocardial ischaemia, SCD and effects on cardiac function [1, 3]. The infant type without adequate collateral filling mostly presents with myocardial infarction or congestive heart failure in the first few years of life. Patients with the adult type have a well-developed collateral system and may present in adult life for the first time with myocardial ischaemia-like complaints, left ventricular dysfunction or SCD [16]. The 2008 ACC/AHA guidelines for the management of adults with congenital heart disease recommend clinical follow-up every 3–5 years after repair [2]. The revised 2018 ACC/AHA and 2020 ESC guidelines do not give recommendations on the follow-up of this patient group [1, 3].

## Coronary arteriovenous fistulas

A CAVF is a communication between a coronary artery and another cardiovascular structure. Regardless of symptoms, large and haemodynamically significant fistulas are corrected, preferentially percutaneously, when they result in cardiac dysfunction. Small or haemodynamically insignificant fistulas are corrected if there are complications; otherwise conservative treatment is an accepted option [2, 3]. The 2008 ACC/AHA guidelines advise that patients visit the outpatient clinic every 3–5 years for evaluation of the haemodynamic sequelae of CAVFs [2]. The 2018 ACC/AHA and 2020 ESC guidelines do not provide recommendations on follow-up [1, 3]. Data on long-term outcomes are scarce, especially in asymptomatic patients with mostly uncorrected CAVFs [1, 2, 17].

## Aims

### Anomalous coronary artery from the opposite sinus

The aim is to optimise our clinical care and create a substantiated protocol from referral to follow-up for patients with ACAOS, especially for cases with an interarterial ACAOS.

### ALCAPA, ARCAPA and CAVF

Follow-up and long-term outcomes are not clearly defined in these patient groups. By maintaining a registry on the follow-up of adult patients, we aim to gain insight into current clinical practice and evaluate the results to provide recommendations for future follow-up of these coronary anomalies.

## Methods

Participating centres of the Multicentre Study on Coronary Anomalies in The Netherlands (MuSCAT) are the Leiden University Medical Centre, University Medical Centre Utrecht, Amsterdam University Medical Centre, Erasmus University Medical Centre, Radboud University Medical Centre and the Medisch Spectrum Twente. The study was approved by the Medical-Ethical Committee Leiden-The Hague-Delft in May 2020.

The study population will consist of consecutive patients with the diagnosis of an ACAOS, ALCAPA, ARCAPA or CAVF. Patient inclusion started in 2020 and will continue for at least 3 years with a minimum of 2 years of follow-up. Patients with a history of (haemodynamically significant) congenital heart defects other than the coronary anomaly and ACAOS patients with proven coronary atherosclerotic disease with significant stenosis will be excluded. After treatment of the stenosis, patients with an ACAOS are reconsidered for inclusion. Due to the rarity of the anomalies under investigation the aim is to include as many patients as possible within the study period, but a sample size target was not set.

### Anomalous coronary artery from the opposite sinus

#### Diagnostics and MuSCAT referral

**Fig. 2:**
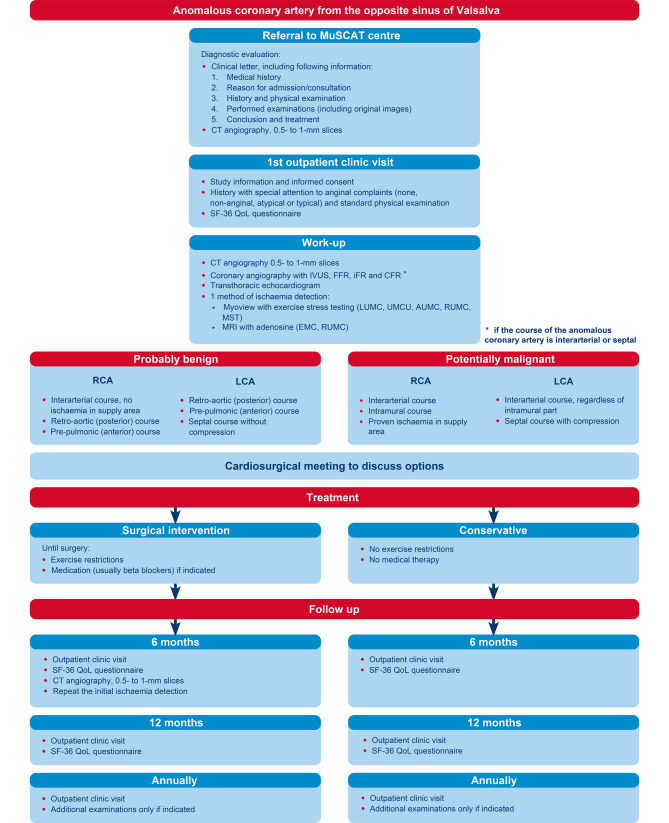
Flowchart of study protocol. *IVUS* intravascular ultrasound, *FFR* fractional flow reserve, *iFR* instantaneous wave-free ratio, *CFR* coronary flow reserve, *LUMC* Leiden University Medical Centre, *UMCU* University Medical Centre Utrecht, *AUMC* Amsterdam University Medical Centre, *EMC* Erasmus University Medical Centre, *RUMC* Radboud University Medical Centre, *MST* Medisch Spectrum Twente, *RCA* right coronary artery, *LCA* left coronary artery, *QoL* quality of life

Fig. 2 shows a flowchart of the study protocol. Patients can be referred via muscat@lumc.nl. After referral, the participating hospital in geographical proximity to the patient is informed, and the patient is referred to that centre for the intake appointment. Written informed consent for participation will be discussed and obtained. On referral, the following information should preferably already be available:

- Referral letter including medical history, reason for admission/consultation, patient history, physical examination findings, results of additional tests, conclusion and treatment strategy.
- Electrocardiogram (ECG)-triggered coronary CT angiography, consisting of 0.5- to 1‑mm slices (0.5 mm preferred), original images and report.
- Results of additional examinations, including original images where applicable.

#### Work-up

After obtaining informed consent, the following examinations are performed in the participating referral centres to evaluate the anatomical and physiological properties of the ACAOS:

- Coronary CT angiography (0.5- to 1‑mm slices) is repeated if prior images are of insufficient quality to adequately assess the (proximal) anatomy of the ACAOS. Coronary anatomy will be described according to the Leiden Convention Coronary Coding system [18, 19].
- CAG with intravascular ultrasound (IVUS) and fractional flow reserve (FFR) measurements (and/or instantaneous wave-free ratio (iFR) or coronary flow reserve (CFR)) of the interarterial and septal ACAOS using adrenaline and adenosine or dobutamine following the protocol previously developed (see Electronic Supplementary Material, Supplement I). On IVUS, ostium morphology (slit-like or ‘cat eye’ appearance) and cross-sectional area stenosis due to compression are evaluated. An FFR value of < 0.8 is considered significant [15]. For iFR a value of < 0.9 and for CFR < 2.0 is considered significant [20, 21].
- Ischaemia detection, mode according to the preference of the referral centre.
- Transthoracic echocardiography to assess ventricular function (and coronary anatomy if possible).
- Quality of life using the SF-36 questionnaire [22, 23].
- Thoracic complaints, classified according to ESC guidelines as typical, atypical or non-anginal [24].

Preferably all examinations are performed as standard of care, as each test addresses a different (patho-)physiological aspect of the ACAOS. All procedures are currently part of the regular care and daily practice in hospitals throughout the Netherlands.

#### Treatment

The treatment strategy is decided by the heart team of the referral centre where the work-up was performed and made according to contemporary clinical care (i.e. not in the context of the study). A MuSCAT working group will retrospectively evaluate per case which treatment they would deem most suitable based on the data available. The MuSCAT working group includes: an adult congenital cardiologist, interventional cardiologists, at least one thoracic surgeon and one European Association of Cardiovascular Imaging level 2 (or equivalent) certified CT cardiologist. This working group will meet (digitally) every 3 months and discuss the study cases. In these meetings advice regarding treatment is generated and the considerations leading to the treatment decision clearly documented so the relevance of every diagnostic examination in the decision-making process can be evaluated. Study team members not from the referral centre will be blinded to the actual treatment strategy chosen. Members of the MuSCAT working group are excluded from evaluation of their own patients to prevent bias. If the MuSCAT working group draws a different conclusion than the heart team of the referral hospital, results are shared if there is still the possibility of changing the treatment strategy. This does not compel the treating cardiologist to follow the advice of the MuSCAT working group. Discrepancies in treatment strategies will be analysed.

#### Follow-up

All patients undergo structural follow-up. Patients with indications for operative treatment are prescribed exercise restriction until surgery. Pharmacological treatment is at the discretion of the treating cardiologist.

##### *Conservative treatment*

Patients with an interarterial ACAOS with proven ischaemia and an indication for surgery who do not wish to undergo an operation are given exercise restrictions for competitive sports and/or maximal exertion and are followed up in the conservative treatment study arm. Asymptomatic patients without evidence of ischaemia will not be placed under any restrictions. Follow-up is structured as follows:

- Six months and 12 months after inclusion: outpatient clinic visit including SF-36 questionnaire and assessment of thoracic complaints.
- After the 12-month visit, annual visits are scheduled and additional examinations performed if indicated.

##### *Surgical intervention*

After surgery, exercise restrictions are waived. Follow-up is structured as follows:

- Six months after surgery: coronary CT angiography (ECG-triggered, 0.5- to 1‑mm slices), ischaemia detection (same test as was performed during the initial work-up), outpatient clinic visit including SF-36 questionnaire and assessment of thoracic complaints.
- Twelve months after surgery: outpatient clinic visit including SF-36 questionnaire and assessment of thoracic complaints.
- After the 12-month visit, annual visits are scheduled and additional examinations performed if indicated.

### ALCAPA, ARCAPA and CAVF

For ALCAPA, ARCAPA and CAVF only a registry is maintained, including clinical follow-up.

## Study outcomes

Primary outcomes are (cardiac) death, myocardial ischaemia attributable to the anomalous vessel, re-intervention after surgical correction of ACAOS and intervention in initially conservatively treated patients. Secondary outcomes are quality of life based on the SF-36 questionnaire, thoracic complaints (typical, atypical, non-anginal) and heart failure requiring medical treatment.

Other outcomes evaluated are the influence of the work-up examinations on treatment choice, the degree of correlation between tests, and differences in treatment decision made by the referral hospital and the MuSCAT working group.

## Conclusion

MuSCAT is the first nationally designed study in patients with ACAOS undergoing a dedicated diagnostic protocol and comparing different diagnostic techniques to evaluate their value in decision making, and also includes a registry of ALCAPA, ARCAPA and CAVF cases. The aim of the study is to provide evidence for protocolling the diagnostic work-up, treatment strategy and follow-up that can be used in daily clinical practice.

## Supplementary Information

Working protocol for CAG with FFR, iFR, CFR and IVUS measurements of the interarterial and septal ACAOS.* Supplement I: protocol coronary angiography with intracoronary measurements*

## References

[1] Stout KK, Daniels CJ, Aboulhosn JA (2019). 2018 AHA/ACC of the American college of cardiology/American heart association task force on clinical practice guidelines. J Am Coll Cardiol.

[2] Warnes CA, Williams RG, Bashore TM (2008). ACC/AHA 2008 guidelines for the management of adults with congenital heart disease: a report of the American college of cardiology/American heart association task force on practice guidelines (writing committee to develop guidelines on the management of adults with congenital heart disease). Developed in collaboration with the American society of echocardiography, heart rhythm society, international society for adult congenital heart disease, society for cardiovascular angiography and interventions, and society of thoracic surgeons. J Am Coll Cardiol.

[3] Baumgartner H, De Backer J, Babu-Narayan SV (2020). 2020 ESC guidelines for the management of adult congenital heart disease. Eur Heart J.

[4] Brothers JA, Frommelt MA, Jaquiss RDB, Myerburg RJ, Fraser CD, Tweddell JS (2017). Expert consensus guidelines: anomalous aortic origin of a coronary artery. J Thorac Cardiovasc Surg.

[5] Grani C, Buechel RR, Kaufmann PA, Kwong RY (2017). Multimodality imaging in individuals with anomalous coronary arteries. JACC Cardiovasc Imaging.

[6] Eckart RE, Scoville SL, Campbell CL (2004). Sudden death in young adults: a 25-year review of autopsies in military recruits. Ann Intern Med.

[7] Harmon KG, Drezner JA, Maleszewski JJ (2014). Pathogeneses of sudden cardiac death in national collegiate athletic association athletes. Circ Arrhythm Electrophysiol.

[8] Maron BJ, Doerer JJ, Haas TS, Tierney DM, Mueller FO (2009). Sudden deaths in young competitive athletes: analysis of 1866 deaths in the United States, 1980–2006. Circulation.

[9] Cheezum MK, Liberthson RR, Shah NR (2017). Anomalous aortic origin of a coronary artery from the inappropriate sinus of Valsalva. J Am Coll Cardiol.

[10] Jegatheeswaran A, Devlin PJ, McCrindle BW (2019). Features associated with myocardial ischemia in anomalous aortic origin of a coronary artery: a congenital heart surgeons’ society study. J Thorac Cardiovasc Surg.

[11] Meyer L, Stubbs B, Fahrenbruch C (2012). Incidence, causes, and survival trends from cardiovascular-related sudden cardiac arrest in children and young adults 0 to 35 years of age: a 30-year review. Circulation.

[12] Grani C, Benz DC, Schmied C (2017). Hybrid CCTA/SPECT myocardial perfusion imaging findings in patients with anomalous origin of coronary arteries from the opposite sinus and suspected concomitant coronary artery disease. J Nucl Cardiol.

[13] Padalino MA, Franchetti N, Hazekamp M (2019). Surgery for anomalous aortic origin of coronary arteries: a multicentre study from the European congenital heart surgeons association. Eur J Cardiothorac Surg.

[14] Padalino MA, Franchetti N, Sarris GE (2019). Anomalous aortic origin of coronary arteries: early results on clinical management from an international multicenter study. Int J Cardiol.

[15] Driesen BW, Warmerdam EG, Sieswerda GT (2018). Anomalous coronary artery originating from the opposite sinus of Valsalva (ACAOS), fractional flow reserve- and intravascular ultrasound-guided management in adult patients. Catheter Cardiovasc Interv.

[16] Regeer MV, Bondarenko O, Zeppenfeld K, Egorova AD (2020). Anomalous left coronary artery from the pulmonary artery: a rare cause of an out-of-hospital cardiac arrest in an adult—a case report. Eur Heart J Case Rep.

[17] Challoumas D, Pericleous A, Dimitrakaki IA, Danelatos C, Dimitrakakis G (2014). Coronary arteriovenous fistulae: a review. Int J Angiol.

[18] Gittenberger-de Groot AC, Koenraadt WMC, Bartelings MM (2018). Coding of coronary arterial origin and branching in congenital heart disease: the modified Leiden convention. J Thorac Cardiovasc Surg.

[19] Koppel CJ, Vliegen HW, Bökenkamp R (2021). The Leiden convention coronary coding system: translation from the surgical to the universal view. Eur Heart J Cardiovasc Imaging.

[20] Escaned J, Echavarría-Pinto M, Garcia-Garcia HM (2015). Prospective assessment of the diagnostic accuracy of instantaneous wave-free ratio to assess coronary stenosis relevance: results of ADVISE II International, Multicenter Study (ADenosine Vasodilator Independent Stenosis Evaluation II). JACC Cardiovasc Interv.

[21] Kern MJ, Lerman A, Bech JW (2006). Physiological assessment of coronary artery disease in the cardiac catheterization laboratory: a scientific statement from the American heart association committee on diagnostic and Interventional cardiac catheterization, council on clinical cardiology. Circulation.

[22] Ware JE, Sherbourne CD (1992). The MOS 36-item short-form health survey (SF-36). I. Conceptual framework and item selection. Med Care.

[23] Aaronson NK, Muller M, Cohen PD (1998). Translation, validation, and norming of the Dutch language version of the SF-36 health survey in community and chronic disease populations. J Clin Epidemiol.

[24] Knuuti J, Wijns W, Saraste A (2019). 2019 ESC guidelines for the diagnosis and management of chronic coronary syndromes: the task force for the diagnosis and management of chronic coronary syndromes of the European society of cardiology (ESC). Eur Heart J.

