# Supplementary material for: The first multicentre study on coronary anomalies in the Netherlands: MuSCAT
Source: Neth Heart J. 2021 Mar 8;29(6):311–7. doi: 10.1007/s12471-021-01556-9 (PMC8160042; doi:10.1007/s12471-021-01556-9)
Supplement: Supplementary file 1 — Working protocol for CAG with FFR, iFR, CFR and IVUS measurements of the interarterial and septal ACAOS. Supplement I: protocol coronary angiography with intracoronary measurements [file 12471_2021_1556_MOESM1_ESM.docx]

**Supplement I: protocol coronary angiography with intracoronary measurements**

**Fractional flow reserve (FFR) and instantaneous wave-free ratio (iFR)**Coronary angiography will be performed according to standard protocol for both FFR and iFR measurements. Often a left guiding catheter is necessary for selective cannulation of the anomalous RCA. For the measurements, a pressure wire is used. FFR is calculated as the ratio of the average distal pressure in the anomalous coronary artery and the aortic pressure measured by the guiding catheter. Maximal hyperaemia is accomplished by intravenous adenosine (140μg/kg/min) or intracoronary adenosine (12-15mg for the RCA and 18-20mg for the LCA). FFR value is considered normal >0.8 (as validated for patients with epicardial coronary artery disease).

The iFR is measured in diastole, beginning 25% into diastole and ending 5ms before the end, and calculated as the ratio of the average distal pressure in the anomalous coronary artery and the aortic pressure. An iFR-value >0.9 is considered normal.

In the case FFR is >0.8, an additional measurement will be done under dobutamine (starting with 5 mg/kg/min with increasing the dosage every 2–3 minutes till a maximum of 50mg/kg/min intravenously) or under adrenaline (starting with 25μg till a maximum dosage of 50μg intravenously). iFR-measurements will also be repeated. Dobutamine and adrenaline are administered until the systolic blood pressure reaches >150mmHg and/or the heart rate reaches >130/min. These additional measurements are done to mimic more naturally the physiological reaction during exercise.

**Coronary flow reserve (CFR)**Pressure bounded CFR can be generated automatically from invasive pressure data on the latest generation of haemodynamic consoles. A CFR value of ≥2.0 is considered normal.

**Intravascular ultrasound (IVUS)**Intravascular ultrasound is done with a mechanical or manual pullback (1mm/sec) after administration of intracoronary nitro-glycerine. Images are scored for the presence of a ‘slit-like ostium’ (oval or ‘cat-eye’ shaped), coronary compression between the aorta and pulmonary artery and atherosclerosis. Also, minimal luminal area (in mm^2^) of the proximal part and the ostium is recorded for each FFR-measurement. Cross-sectional area stenosis will be calculated as [100 x (reference lumen area – minimal lumen area)/reference lumen area].
